# Origin of Carbon and Essential Fatty Acids in Higher Trophic Level Fish in Headwater Stream Food Webs

**DOI:** 10.3390/biom9090487

**Published:** 2019-09-13

**Authors:** Megumu Fujibayashi, Yoshie Miura, Reina Suganuma, Shinji Takahashi, Takashi Sakamaki, Naoyuki Miyata, So Kazama

**Affiliations:** 1 Faculty of Bioresource Sciences, Akita Prefectural University, Kaidobata-Nishi 241-438, Shimoshinjo Nakano, Akita city, Akita Prefecture 010-0195, Japan; 2 Department of Urban and Environmental Engineering, Faculty of Engineering, Kyushu University, 744, Motooka, Nishiku, Fukuoka 819-0395, Japan; 3 Technical Division, School of Engineering, Tohoku University, 6-6-06 Aoba, Aoba, Sendai, Miyagi 980-8579, Japan; 4 Department of Civil and Environmental Engineering, School of Engineering, Tohoku University, 6-6-06 Aoba, Aoba, Sendai, Miyagi 980-8579, Japan

**Keywords:** fatty acids, dietary sources, allochthonous, *Salvelinus leucomaenis*

## Abstract

Dietary carbon sources in headwater stream food webs are divided into allochthonous and autochthonous organic matters. We hypothesized that: 1) the dietary allochthonous contribution for fish in headwater stream food webs positively relate with canopy cover; and 2) essential fatty acids originate from autochthonous organic matter regardless of canopy covers, because essential fatty acids, such as 20:5ω3 and 22:6ω3, are normally absent in allochthonous organic matters. We investigated predatory fish *Salvelinus leucomaenis* stomach contents in four headwater stream systems, which are located in subarctic region in northern Japan. In addition, stable carbon and nitrogen isotope ratios, fatty acid profile, and stable carbon isotope ratios of essential fatty acids were analyzed. Bulk stable carbon analysis showed the major contribution of autochthonous sources to assimilated carbon in *S. leucomaenis.* Surface baits in the stomach had intermediate stable carbon isotope ratios between autochthonous and allochthonous organic matter, indicating aquatic carbon was partly assimilated by surface baits. Stable carbon isotope ratios of essential fatty acids showed a positive relationship between autochthonous sources and *S. leucomaenis* across four study sites. This study demonstrated that the main supplier of dietary carbon and essential fatty acids was autochthonous organic matter even in headwater stream ecosystems under high canopy cover.

## 1. Introduction

Aquatic animals are supported by two basal organic carbon sources, autochthonous (aquatic primary producers) and allochthonous sources (fallen leaf litter and insects from surrounding terrestrial ecosystems) [1]. Contributions of these basal organic carbon sources to aquatic food webs depend on the proportion of microalgal and phytoplankton productivity, and the number of terrestrial subsidies [2]. For lower order headwater streams, as high canopy cover promotes abundant inputs of litter falls [3] and limited productivity of attached algae due to shading effects [4], the main carbon sources for aquatic consumers are predicted as allochthonous by the river continuum concept (RCC) [5]. However, food web studies in headwater streams have demonstrated that the dominant dietary contribution for macroinvertebrates is both autochthonous [6,7] and allochthonous [8,9], indicating that the predominant carbon sources for headwater stream food webs are unclear. 

Previous studies on the dietary contribution of these basal organic carbon sources have mainly focused on quantitative contribution; however, studies focusing on dietary quality are relatively rare in headwater stream food webs [10,11]. For instance, essential fatty acids are known to be important nutrition for fish health [12,13,14]. In particular, the roles of 20:5ω3 and 22:6ω3 in fish growth, survival, and reproduction have been studied in many species [15,16,17]. These studies have demonstrated that dietary essential fatty acids could improve fish condition. Freshwater fish can synthesize 20:5ω3 and 22:6ω3 if 18:3ω3, which is precursor of these two essential fatty acids, is available from dietary sources [13]. However, the conversion efficiency in aquatic animals is generally very low. Consequently, direct intake of these fatty acids from dietary sources is required [18]. However, terrestrial organic matter contains only 18:3ω3 but not 20:5ω3 and 22:6ω3, indicating that terrestrial organic matter is nutritionally poor [18]. 20:5ω3 is present at high levels in diatoms [19], which are sometimes the dominant algae attached to the surface of substrates in headstream ecosystems [20]. This implies that autochthonous organic carbon (i.e., attached algae) may be the main essential fatty acid source for consumers, although other carbon components are derived from allochthonous inputs in headwater streams.

Stable isotope ratios of bulk carbon in consumers reflect those of assimilated diet with only minor fractionation (<1‰), which enable us to infer its dietary carbon sources [21]. In addition, stable carbon isotope ratios of autochthonous organic sources (e.g., attached algae) and allochthonous organic sources are distinguishable in many cases [8,22]. Accordingly, the contribution of allochthonous organic matter to stream food webs has been evaluated by bulk carbon stable isotope ratios [8]. Stable isotope ratios of bulk nitrogen (δ^15^N) have also been used in food web studies, because of its usefulness to evaluate the trophic position of animals owing to the substantial enrichment of approximately 3‰ relative to that of assimilated diet [23,24,25].

For tracing the dietary essential fatty acid origin, although their carbon stable isotope ratios would be helpful, information about isotopic fractionation of essential fatty acids between diet and consumers is limited. For instance, Budge et al. [26] demonstrated that there was no isotopic fractionation in several essential fatty acids between the diet and serum of fish in a feeding experiment with Atlantic Pollock. Moreover, an almost isotopically unchanged transfer of 18:2ω6 and 18:3ω3 between diet and zebrafish *Danio rerio* was observed in a 100-day feeding experiment with constant dietary sources [27]. Several aquatic food web studies have already assumed no fractionation of isotopic value of essential fatty acids [28,29]. On the contrary Gradyshev et al. [30] found gradual depletion of stable carbon isotope ratios of essential fatty acids, including 18:2ω6 and 18:3ω3, through higher trophic levels in a stream food chain, suggesting that fractionation was negative. Depleted fractionation in 18:3ω3 was also reported in zooplankton in a feeding experiment [31]. Depleted fractionation in other essential fatty acids have been also reported by a feeding experiments with Daphnia [32]. This can be explained by lighter compounds being assimilated preferentially. As above, the information on fractionation of fatty acids are not sufficient and they were conflicting (i.e., no or small fractionation and negative fractionation). Nielsen et al. [33] pointed out more information on the fractionation of fatty acids are required for diet tracing study. Thus, we did not apply a constant value of isotopic fractionation of essential fatty acids in a food chain in this study. We applied a correlation analysis of stable carbon isotope ratios of essential fatty acids between diet and consumer among the study sites. If organic sources consistently contribute to consumers and have wider isotopic differences, one would expect to detect a significant and positive relationship in stable carbon isotope ratios of essential fatty acids between consumers and assimilated food source regardless of isotopic fractionation [34,35,36].

Here, we tested the following two hypotheses that: 1) the dietary allochthonous contribution for fish in headwater stream food webs positively relates with canopy cover; and 2) essential fatty acids originate from autochthonous organic matter regardless of canopy covers. To test these hypotheses, we analyzed fatty acid compositions and bulk carbon and compound-specific isotope ratios in fish from four headstream ecosystems. 

## 2. Materials and Methods

### 2.1. Sampling

We conducted field surveys in four headwater streams, located in subarctic area in the northern part of Japan from July to September 2016 (Table 1). Canopy cover was calculated from a hemispherical photography taken from the center of each stream using CanopOn2 program [37].

*Salvelinus leucomaenis* is a dominant predatory fish in these four study sites. The main dietary sources of *S. leucomaenis* are larvae and adults of aquatic insects and terrestrial insects [38,39]. *S. leucomaenis* was sampled by fishing. The total length and whole wet weight were measured and a muscle near the pelvic fins was dissected for further analyses. The stomach was preserved in 90% ethanol. For autochthonous organic sources analyses, epilithic biofilm, which was mainly composed of attached algae, was removed using a brush from several randomly selected stones. The collected epilithic biofilm was placed in a plastic sampling bottle with distilled water. The bottle containing algae was filtered through two glass filters (GFF; Whatman, Little Chalfont, UK) in the laboratory for fatty acid and bulk stable carbon and nitrogen isotope analyses. For allochthonous organic sources analyses, decomposed immersed leaf litter was sampled into a plastic bag. Both autochthonous and allochthonous organic sources were sampled in triplicate. The larvae of aquatic insects including Ephemeroptera, Trichoptera, and Plecoptera, which are potentially a direct dietary source for *S. leucomaenis*, were collected using D-frame nets (250 µm mesh) and sorted in the laboratory. Heptageniidae and Ephemerellidae were used for further analysis as they were dominant and commonly detected across all four study sites. All collected samples were transported to the laboratory in a cooler box. All samples were placed in a plastic bag separately and stored in a freezer at −20 °C until further analysis. 

Additional sampling for analyses of bulk stable isotope ratios of *S. leucomaenis*, its stomach contents, epilithic biofilms, and leaf litter were conducted in the same location of Babame in July 2018. The same sampling procedure was applied except for stomach contents. The stomach of *S. leucomaenis* was placed in a plastic bottle with distilled water and moved to laboratory in a cooler. The stomach contents were identified and separated. *S. leucomaenis*, epilithic biofilms, and leaf litter were treated following the method mentioned above. All samples were preserved in a plastic bag and stored in a freezer at −20 °C until further analysis.

### 2.2. Analyses 

The stomach contents of each *S. leucomaenis* individual were divided into four groups based on the morphological characteristic using a stereoscopic microscope: water baits (larvae of aquatic insects) and surface baits (adults of aquatic insects and terrestrial insects) according to the definition of Tsuda [38], terrestrial plants, and unknown. Each group was weighted and the contribution of each group was calculated. 

Freeze-dried samples of *S. leucomaenis*, both organic sources, and aquatic insects were used for the ‘one-step method’ [40] for fatty acid analysis. For aquatic insects, two or three individuals were pooled as one sample, and prepared three samples in each study sites. Freeze-dried samples were moved to a 10 mL glass tube. For *S. leucomaenis,* aquatic insects, and leaf litter, approximately 50 mg of homogenized sample was used. For epilithic biofilms, one sheet of GFF was used in the analysis. One milliliter of an internal standard (0.1 mg of tricosanoic acid per 1 mL of hexane), 1 mL of hexane, and 0.8 mL of 14% boron trifluoride methanol were added to the 10 mL glass tube. Nitrogen gas was then used to fill the head space. The glass tube was placed in a 100 °C dry bath for 2 h, followed by cooling to room temperature, and 0.5 mL of hexane and 1 mL of ultrapure water were added. The glass tube was vigorously shaken manually and centrifuged for 3 min at 2,500 rpm. The upper layer of hexane, containing fatty acid methyl esters (FAMEs), was transferred to a 1.5 mL gas chromatography (GC) vial. Solid residues of *S. leucomaenis* were used for bulk carbon and nitrogen isotope ratio analysis. 

One microliter of FAME solution was injected in a gas chromatograph (Trace GC, Thermo Fisher Scientific, Bremen, Germany) equipped with a capillary column (Select FAME, 100 m × 0.25 mm i.d.; Agilent Technologies, Santa Clara, CA, USA). The GC analysis was carried out under the analytical conditions described by Fujibayashi et al. [28]. Each fatty acid peak was identified by comparing their retention times with those of commercial authentic standard mixtures (Supelco, Inc., Bellefonte, PA, USA). The peak area was used for calculating the contribution of each fatty acid to total fatty acids. 

After fatty acid analysis by GC, the remaining hexane sample was used to analyze the essential fatty acids stable carbon isotope ratio. FAMEs in the hexane solution were injected into a GC–isotope ratio mass spectroscopy instrument (Trace GC Ultra/Delta-V Advantage; Thermo Fisher Scientific, Bremen, Germany), which was equipped with a capillary column (SP2560, 100 m × 0.25 mm i.d.; Supelco, Inc., Bellefonte, PA, USA). The operating conditions were as described by Fujibayashi et al. [29]. Each essential fatty acid peak was identified as described above for the GC-FID analysis. Stable carbon isotope ratios of fatty acids were determined using the following formula:

δ^13^C or N (‰) = (R_sample_/R_standard_ − 1) × 1000 (1)

where R_sample_ is the ^13^C/^12^C ratio of the sample, and R_standard_ is the ^13^C/^12^C ratio of the international isotopic standard (i.e., Vienna Pee Dee Belemnite).

Correction for the effect of additional carbon from boron trifluoride methanol on δ^13^C was conducted according to Fujibayashi et al. [29]. The stable carbon isotopes of fatty acids in *S. leucomaenis*, epilithic biofilms, and terrestrial litter samples were analyzed.

Dried solid residues of *S. leucomaenis*, subsamples of freeze-dried terrestrial litter, GFFs (epilithic biofilms), and aquatic insects were used for bulk stable carbon and nitrogen isotope ratio analysis. Utilization of solid residues after a one-step method potentially changes the isotopic value. Therefore, the relationship between the stable isotope ratios of carbon and nitrogen in original samples and those in the corresponding dried solid residue after the one-step method were checked in advance with freshwater fish muscle samples (Supplementary file Figure S1). For nitrogen, while a significant positive relationship was detected, variation was relatively high. Thus, estimation of trophic position of *S. leucomaenis* using solid resides may include some extents of error. However, stable carbon isotope rations of solid residues well reflected that of the original samples, and the utilization of solid resides for stable carbon isotope analysis was applied in this study. For the sampling of aquatic insects, one individual was used for one sample, and we prepared three samples for each study site. All samples were weighed in microcapsules and injected into an elemental analyzer (Flash EA; Thermo Fisher Scientific, Bremen, Germany) linked to a mass spectrometer (Delta-V Advantage; Thermo Fisher Scientific, Bremen, Germany). Stable isotope ratios of bulk carbon and nitrogen were expressed as Equation (1); where R_sample_ is the ^13^C/^12^C or ^15^N/^14^N ratio of the sample, and R_standard_ is the ^13^C/^12^C and the ^15^N/^14^N ratio of the international isotopic standard (Vienna Pee Dee Belemnite, and atmospheric N_2_, respectively). 

## 3. Results

Although canopy cover was high in both the first order rivers, Babame and Kurikoma, with 93.5% and 91.1%, respectively; the third-order rivers, Hayakuchi and Naruse, had relatively open canopy with 67.6% and 63.5%, respectively. 

**Figure 1:**
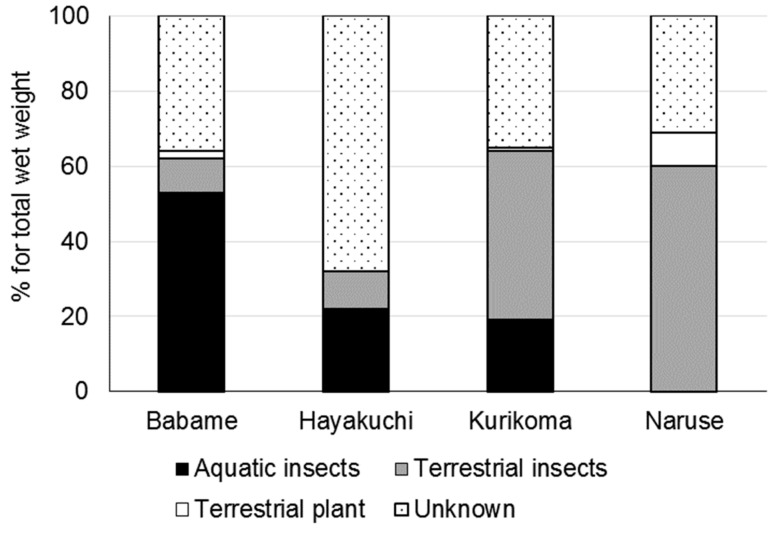
Stomach contents (wet weight %) of *S. leucomaenis* from four study sites.

Ten and 11 individuals of *S*. *leucomaenis* were caught in Babame and Kurikoma by fishing; however, just one individual was caught in Hayakuchi and Naruse. There was a relatively high proportion, ranging from 31% to 68%, of unknown components in the stomach contents that could not be identified because of decomposition (Figure 1). Terrestrial plants were almost not detected in the stomach contents, while water and surface baits were dominant in the stomach contents in *S*. *leucomaenis*. There was no obvious relationship between water and surface bait contribution and canopy cover.

**Figure 2:**
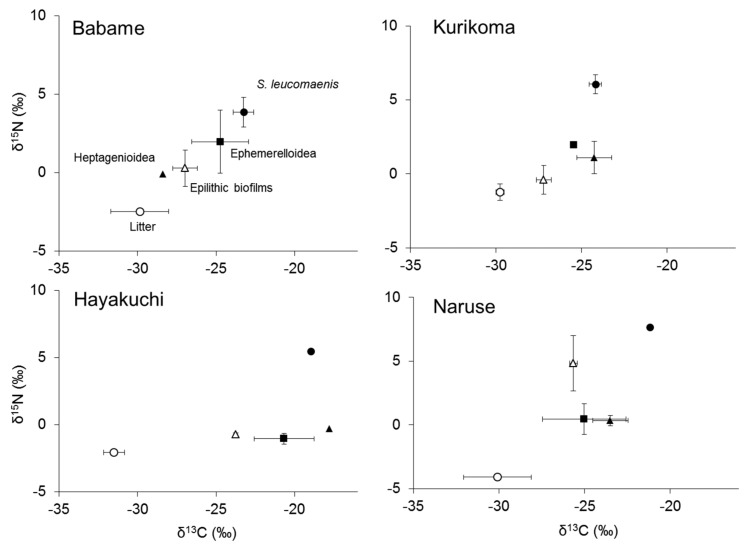
Stable isotope ratios biplot for bulk carbon and nitrogen in basal organic carbon sources and consumers in four study sites. Error bars represent standard deviation.

Although stable carbon and nitrogen isotope ratios of *S*. *leucomaenis* varied among study sites, the trophic position of *S*. *leucomaenis* was always the highest (Figure 2). Aquatic insects were generally at a lower position than *S. leucomaenis* with similar carbon isotopic values. Leaf litter showed the most depleted isotopic value for both carbon and nitrogen in all study sites. The range of stable carbon isotope ratios of leaf litter was relatively narrow, from −31.5‰ in Hayakuchi to −29.8‰ in Kurikoma. For epilithic biofilms, stable carbon isotope ratios were more enriched than terrestrial litter and showed a wider range, from −27.2‰ in Kurikoma to −23.8‰ in Hayakuchi. 

**Figure 3:**
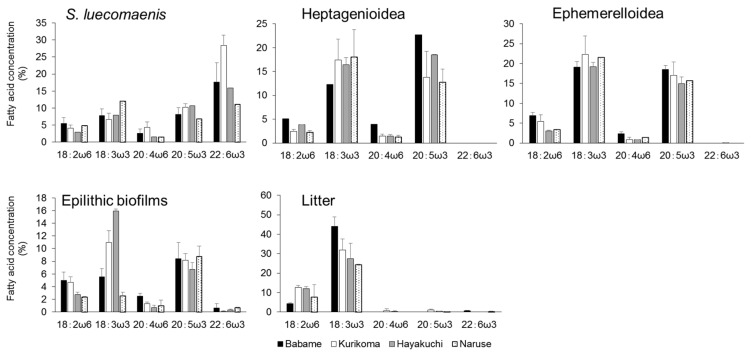
Contribution of essential fatty acids in basal organic carbon sources and consumers in the four study sites. Error bars represent standard deviation.

All essential fatty acids were detected in *S. leucomaenis* (Figure 3). The major essential fatty acid was 22:6ω3. The average contribution of 22:6ω3 was the highest in *S. leucomaenis* from Kurikoma. With respect to other essential fatty acids, the other omega-3 fatty acids, such as 18:3ω3 and 20:5ω3, presented a higher contribution than that of omega-6 fatty acids. In both aquatic insects, essential fatty acid distribution was similar, with no 22:6ω3. The major fatty acids in both aquatic insects were 18:3ω3 and 20:5ω3. This essential fatty acid pattern was similar among study sites. Epilithic biofilms mainly consisted of 18:3ω3 and 20:5ω3. The contribution of 20:5ω3 was relatively constant in all study sites, while 18:3ω3 contribution varied widely among study sites. Only small amounts of 22:6ω3 were detected from epilithic biofilms. Terrestrial litter only contained 18:2ω6 and 18:3ω3. Other C20 essential fatty acids were only detected at low percentages.

**Figure 4:**
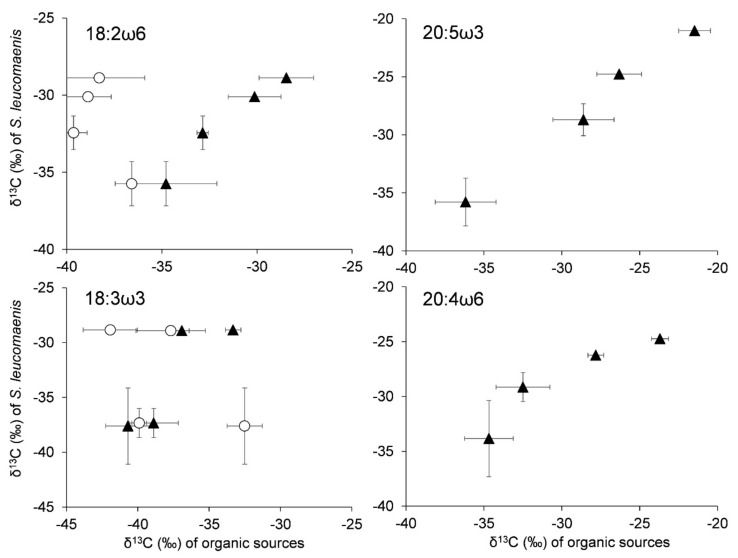
Relationship between stable isotope ratios of essential fatty acid in *S. leucomaenis* and basal organic sources from the four study sites. The black triangle and open circle represent autochthonous organic sources (epilithic biofilms) and allochthonous organic sources (leaf litters), respectively. Error bars represent standard deviation.

The stable carbon isotope ratios of 18:2ω6, 20:4ω6, and 20:5ω3 in *S. leucomaenis* and epilithic biofilms were almost the same across study sites (Supplementary file Figure S2); consequently, a significant or marginally positive relationship was detected between them (correlation analysis: 18:2ω6, n = 4, r = 0.98, *p* < 0.01; 20:5ω3, n = 4, r = 0.99, *p* < 0.001; 20:4ω6, n = 4, r = 0.94, *p* = 0.063) (Figure 4). Although there was no statistical significance, a positive trend was detected between the stable carbon isotope ratios of 18:3ω3 in epilithic biofilms and that in *S. leucomaenis*. The stable carbon isotope ratios of 18:3ω3 were generally lower in the epilithic biofilms than in *S. leucomaenis* (Supplementary file Figure S2). Contrarily, the stable carbon isotope ratios of 18:2ω6 and 18:3ω3 in leaf litter were not positively correlated with those of in *S. leucomaenis*. 

**Figure 5:**
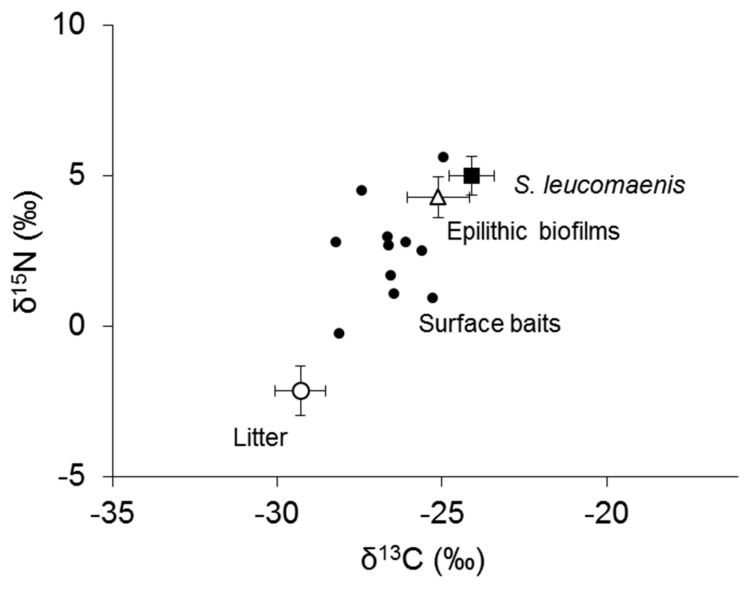
Biplot for stable isotope ratios of bulk carbon and nitrogen of basal organic carbon sources, *S. leucomaenis,* and surface baits from *S. leucomaenis* stomachs in Babame, 2018. Error bars represent standard deviation.

The bulk carbon and nitrogen isotope ratios of leaf litter in Babame in July 2018 showed values similar to those in July 2016, −29.3‰ for carbon, and −2.1‰ for nitrogen (Figure 5). The carbon and nitrogen stable isotope ratios of epilithic biofilms were −25.1‰ and 4.3‰, respectively. Terrestrial insects in the stomach of *S. leucomaenis* were between leaf litters and epilithic biofilms for carbon and nitrogen stable isotope ratios, with a mean value of −26.7‰ for carbon and 2.2‰ for nitrogen. The bulk stable carbon and nitrogen isotope ratios of *S. leucomaenis* were the most enriched among all samples, and close to those of epilithic biofilms.

## 4. Discussion

### 4.1. Origin of Organic Sources

Canopy cover is an important factor for biogeochemical and biological processes in headwater streams [41]. High leaf litter input and limited primary production are expected in headwater streams. Therefore, a positive relationship between allochthonous contribution and canopy cover was expected [42]. For instance, dietary inputs of surface baits, such as emerged aquatic insects and terrestrial insects, can be expected to increase as the canopy cover increased. However, the stomach contents did not show the expected patterns. Furthermore, our results of bulk carbon stable isotope ratios demonstrated that allochthonous contribution was very rare in *S. leucomaenis*, even in the Babame and Kurikoma study sites, where the canopy cover was very high (>90%). The isotopic positions of *S. leucomaenis* were relatively close to aquatic insects for carbon and higher for nitrogen, indicating that aquatic insects were diet items for *S. leucomaenis*. There were isotopic differences of 2–3‰ in carbon between epilithic biofilms and aquatic insects. The differences seem to be relatively high if a dietary relationship was assumed between epilithic biofilms and aquatic insects, because 0–1‰ fractionations have generally been assumed [21]. These relatively high differences may be explained under some assumptions. First, epilithic biofilms are the mixture of various organic sources with not only algal species, but also terrestrial organic matter [43]. According to our fatty acid analysis of epilithic biofilms, 24:0, which is a fatty acid biomarker of higher plants [44], was detected at 1–2% (data not shown), indicating that the analyzed epilithic biofilms contained terrestrial organic matter. Therefore, the stable carbon isotope ratios are the average of all contained organic matter [43]. The stable carbon isotope ratios of leaf litter showed generally low values in our study sites, indicating pure attached algae stable isotope values were likely more enriched than the analyzed values. If aquatic grazers can selectively utilize specific preferred carbon sources from periphyton [45], the observed wider fractionation between aquatic insects and epilithic biofilms is explainable. This wider fractionation could also be explained by temporal variation of stable carbon isotope ratios in epilithic biofilms. Although the leaf litter stable carbon isotope ratios were relatively constant among the study sites, those of epilithic biofilms widely varied, even in the same study sites between 2016 and 2018 in Babame. It is known that algal stable carbon ratios varied along the gradient of some environmental factors such as isotopic value of dissolved inorganic carbon [8,46] and growth stage [46,47]. This potentially high variability made it difficult to infer the algae dietary contribution for consumers under one-time sampling of stable carbon isotope. Contrastingly, stable carbon isotope ratios of animals were considered to integrate relatively long times [22]. The stable carbon isotope ratios of aquatic insects were generally higher than those of leaf litter and relatively similar to those of attached algae. Thus, the main organic source for *S. leucomaenis* seems to be autochthonous, that is, epilithic biofilms transferred through aquatic insects, regardless of canopy cover in the study sites.

Major dietary contribution of autochthonous sources for headstream consumers has been reported for tropical [6], subtropical [48], and temperate regions [7]. For instance, Lewis et al. [49] showed a major contribution of autochthonous dietary input even under the dominant input of allochthonous organic matters. These contrary observations against the predictions of RCC can be attributed to poor food quality of terrestrial organic matters and high quality of algae [18,50]. However, as observed from the stomach contents analysis, there was a substantial contribution of fallen insects to *S. leucomaenis* diet, in accordance to previous studies on *S. leucomaenis* stomach content [51]. In this study, we could not identify each species. However, according to the stable carbon isotope ratio analysis of surface baits in stomach contents, the carbon sources of these surface baits must have been derived partly from aquatic algae. Some riparian insects spend larval life in aquatic ecosystems and have been known to play an important role transferring highly unsaturated fatty acids from aquatic to terrestrial ecosystems by emerging [52]. This indicated that parts of surface baits can also be a vector of autochthonous carbon going back to aquatic ecosystems. Several researchers pointed out that dietary utilization of riparian insects is one of the pathways to acquire autochthonous organic sources [18]. Therefore, to evaluate organic matter origin, assimilation-based methods (e.g., stable isotope and fatty acids) are required.

### 4.2. Origin of Essential Fatty Acids

It is well known that lipids have more negative δ^13^C values than that of other biochemical compounds because lighter carbons tend to be used for conversion of pyruvate to acetyl coenzyme A in lipid synthesis [53]. Therefore, several studies have reported more depleted isotopic values in essential fatty acids than that of bulk carbon [27,30]. Similarly, stable isotope ratios of essential fatty acids were substantially depleted compared with that of bulk carbon in the current study. Furthermore, spatial difference was also observed in both bulk and essential fatty acids isotope. Enriched isotopic value was observed in Hayakuchi and Naruse Rivers where canopy cover was relatively low, indicating high availability of sunlight for photosynthesis. It is known that high growth rate makes algal isotopic value enriched due to the increase of contribution of heavy CO_2_ [46]. The observed wider difference in isotopic ratios of epilithic biofilm can be reflected in the photosynthetic activity in each study site.

*S. leucomaenis* contained all essential fatty acids. 18:2ω6 and 18:3ω3 were detected in both organic carbon sources, leaf litters and epilithic biofilms. These fatty acids are not synthesized by animals [54], indicating the origin of these fatty acids in *S. leucomaenis* was either or both of them. We observed a significant positive relationship of stable carbon isotope ratios of 18:2ω6 between epilithic biofilms and *S. leucomaenis* indicating that this essential fatty acids in *S. leucomaenis* was mainly of autochthonous origin. For 18:3ω3, although a positive tendency was observed between epilithic biofilms and *S. leucomaenis*, this relationship was not statistically significant. However, since 18:3ω3 cannot be synthesized by *S. leucomaenis*, 18:3ω3 must come from either epilithic biofilms or leaf litter. Epilithic biofilms seem to be a probable candidate for the origin in 18:3ω3 for *S. leucomaenis* because isotopic values of leaf litter were highly depleted compared to that of *S. leucomaenis*, which cannot be explained by the previously reported isotopic fractionation, small [27] or depleted and inconsistent [32].

The origin of 20:5ω3 and 20:4ω6 seems to be epilithic biofilms or biosynthesis from their corresponding precursors, namely 18:3ω3 and 18:2ω6, respectively [13]. The stable carbon isotope ratios of both essential fatty acids showed positive relationships between *S. leucomaenis* and epilithic biofilm, indicating that these essential fatty acids were also of autochthonous origin. If we assume the origin of essential fatty acids to be epilithic biofilm, isotopic fractionation through two trophic levels, namely epilithic biofilm, aquatic insects, and *S. leucomaenis* for 18:2ω6, 20:5ω3, and 20:4ω6 was −0.9–0.4‰, 0–1.5‰, and −1.0–3.3‰, respectively. On the contrary, for 18:3ω3, the expected isotopic fractionation of epilithic biofilm to *S. leucomaenis* via aquatic insects was 1.6–8.0‰. Fujibayashi et al. [29] found no significant difference of isotopic value of 18:3ω3 between freshwater fish and blooming cyanobacteria. However, the mechanism of this inconsistent and variable fractionation for 18:3ω3 was not explainable in this study. Further research is required for isotopic fractionation of essential fatty acids in food chains. 

While 22:6ω3 was the most abundant essential fatty in *S. leucomaenis*, both organic sources and aquatic insects did not contain 22:6ω3. However, we only analyzed the fatty acid content in two ephemeral groups. Moreover, the absence or very small contribution of 22:6ω3 in aquatic insects has been reported for a wide range of aquatic insect taxa [55,56,57]. As 22:6ω3 was less available from dietary sources, 22:6ω3 detected in *S. leucomaenis* must be biosynthesized from its precursor [11]. During elongation from 20:5ω3 to 22:6ω3, lighter carbon may be preferentially added. Consequently, 22:6ω3 isotopic value depleted compared with that of 20:5ω3 [27]. However, we found that almost the same or slightly enriched isotopic values in 22:6ω3 compared with that of 20:5ω3 (Supplementary file Figure S2). The same tendency was observed in several aquatic consumers, including fish, in Yenisei River [30]. Gladyshev et al. [30] pointed out the possibility that the acetate pool, which is required for elongation of fatty acid, is significantly enriched in ^13^C compared with fatty acids. While further study is needed to comprehensively understand essential fatty acid dynamics in aquatic ecosystems, our results demonstrated that the main source of essential fatty acids in headstream food webs was autochthonous organic matter. 

## 5. Conclusions

Dietary origin of total organic carbon and essential fatty acids for the predatory fish *S. leucomaenis* was investigated in the four headwater streams with the two hypotheses: (1) the dietary allochthonous contribution for fish in headwater stream food webs positively relate with canopy cover; and (2) essential fatty acids originate from autochthonous organic matter regardless of canopy cover. Our results indicated that autochthonous organic matters were the main dietary origin of not only essential fatty acids, but also total organic carbon regardless of canopy cover. 

## Figures and Tables

**Table 1:** Description of study sites in this study.

Study Site	GPS	Order	Sampling Date	Canopy (%)	Water Temperature (°C)
Babame	N39.8678°, E140.2552°	1	10 July	93.5	15.9
Hayakuchi	N40.4227°, E140.3470°	3	20 July	67.6	15.6
Kurikoma	N38.9169°, E140.7356°	1	2 September	91.1	14.2
Naruse	N 39.0716°, E 140.7187°	3	18 September	63.5	18.5

## Supplementary Materials

The following are available online at https://www.mdpi.com/2218-273X/9/9/487/s1. Figure S1 Relationship between stable isotope ratios of bulk and lipid free fish muscle samples (n = 137). Figure S2 Isotopic value of essential fatty acids in collected samples from each study stream. Error bars represent standard deviation.
