# Supplementary material for: Origin of Carbon and Essential Fatty Acids in Higher Trophic Level Fish in Headwater Stream Food Webs
Source: Biomolecules. 2019 Sep 13;9(9):487. doi: 10.3390/biom9090487 (PMC6770133; doi:10.3390/biom9090487)
Supplement: Supplementary file 1 [file biomolecules-09-00487-s001.pdf]

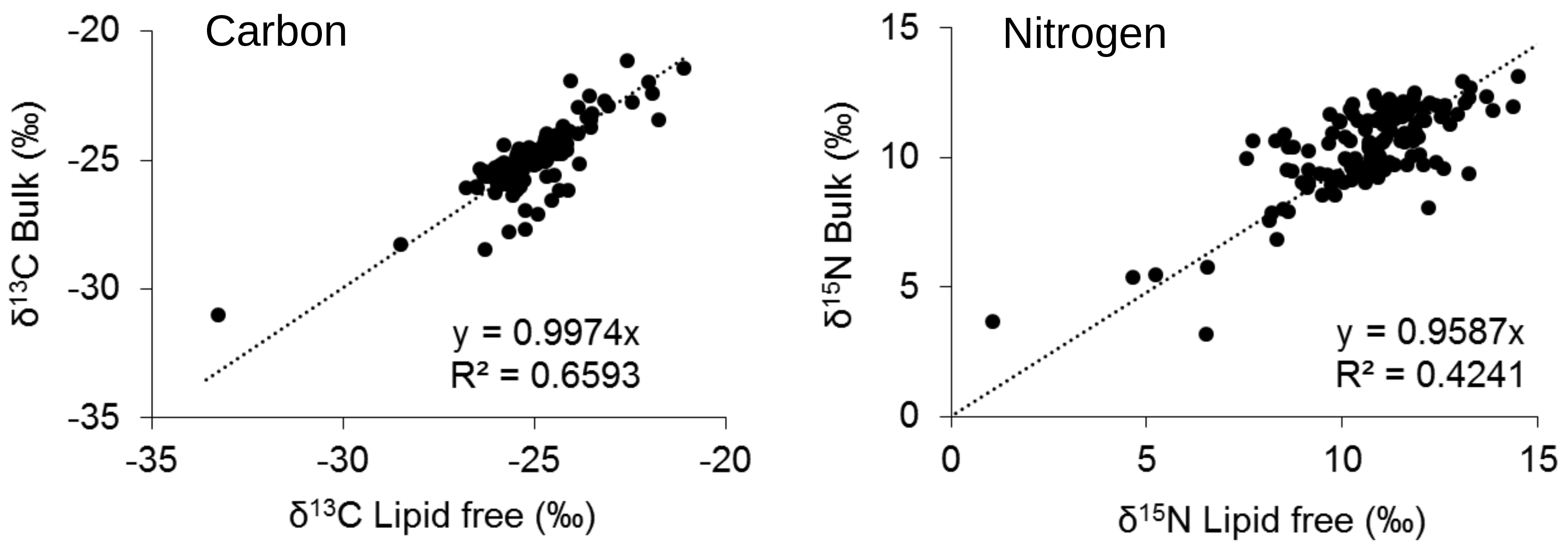

Supplementary file

Figure S1

The relationship between stable isotope ratios of bulk and lipid free fish muscle samples (n = 137).

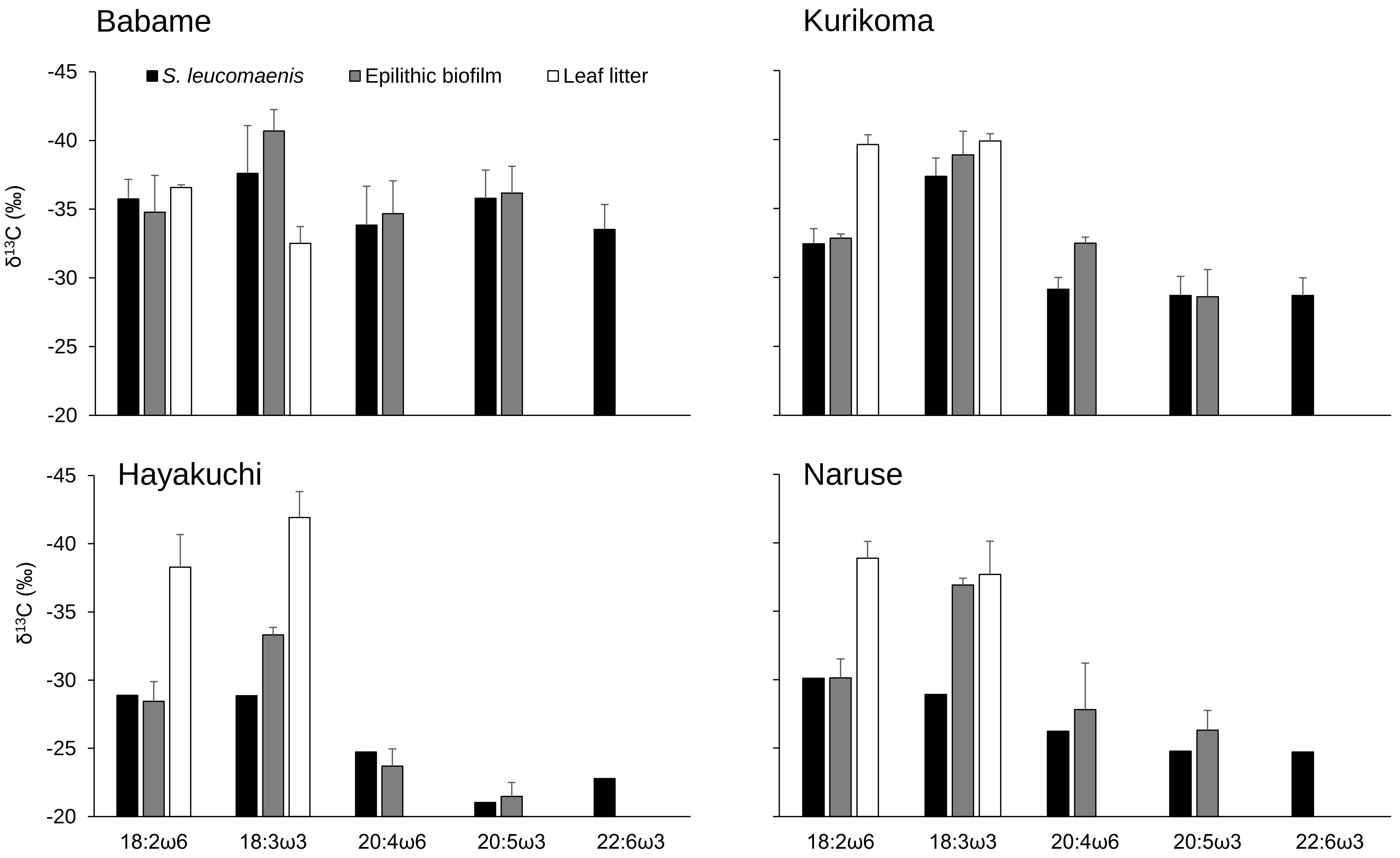

Figure S2 Isotopic value of essential fatty acids in collected samples from each study stream. Error bars represent standard deviation.
